# Plasma Cell Vulvitis Treated with Platelet-rich Plasma: A Case Report

**DOI:** 10.31729/jnma.7622

**Published:** 2022-08-31

**Authors:** Paras Oli, Harihar Adhikari, Deeptara Pathak Thapa

**Affiliations:** 1 Nepal Medical College and Teaching Hospital, Jorpati, Kathmandu, Nepal; 2 Department of Dermatology, Nepal Medical College and Teaching Hospital, Jorpati, Kathmandu, Nepal

**Keywords:** *case report*, *immunosuppressant*, *platelet-rich plasma*, *vulvitis*

## Abstract

Plasma cell vulvitis is a rare inflammatory disorder of the vulva with an unknown aetiology, characterised by mucosal inflammation. It commonly manifests as pain, itching, dyspareunia, and dysuria and clinically presents as erythematous plaque and macules on the vulva. This condition is refractory to available treatment modalities in the literature. We present a case of a 70-year-old female with histopathologically proven plasma cell vulvitis treated by platelet-rich plasma therapy after multiple failed treatment attempts with topical steroids and immunomodulators. The patient improved both symptomatically and clinically on follow-up with platelet-rich plasma therapy. Platelet-rich plasma which is a new novel treatment can be a therapeutic option for recalcitrant cases of plasma cell vulvitis.

## INTRODUCTION

Plasma cell vulvitis, known as Zoon vulvitis, is a rare idiopathic chronic benign inflammatory disorder of the vulvar mucosa. It has features similar to diseases like lichen planus, and squamous cell carcinoma. Though it is mostly asymptomatic, it may present with pain, itching, dyspareunia, and dysuria. The therapeutic options for this disease are limited.^1^ We report the first instance of a rare case of plasma cell vulvitis from Nepal treated successfully with platelet-rich plasma (PRP) after other treatment modalities had failed.

## CASE REPORT

**Figure 1:**
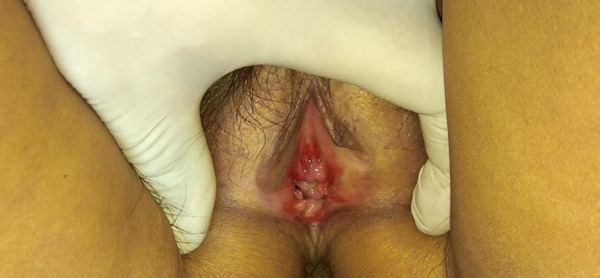
Erythematous plaque in the vestibule and periurethral region (before treatment).

A 70-year-old female presented to our outpatient department with a history of reddish lesions on the vulva which were associated with itchiness, pain, and irritation on and off for the last 3 years. She had visited many centres and taken multiple treatments like topical corticosteroid and topical tacrolimus, but there was no improvement. She was suffering a lot due to her health condition. There was an absence of similar symptoms in her family. On clinical examination, there were multiple shiny erythematous plaques in the vulva vestibule and the periurethral area (Figure 1).

**Figure 2:**
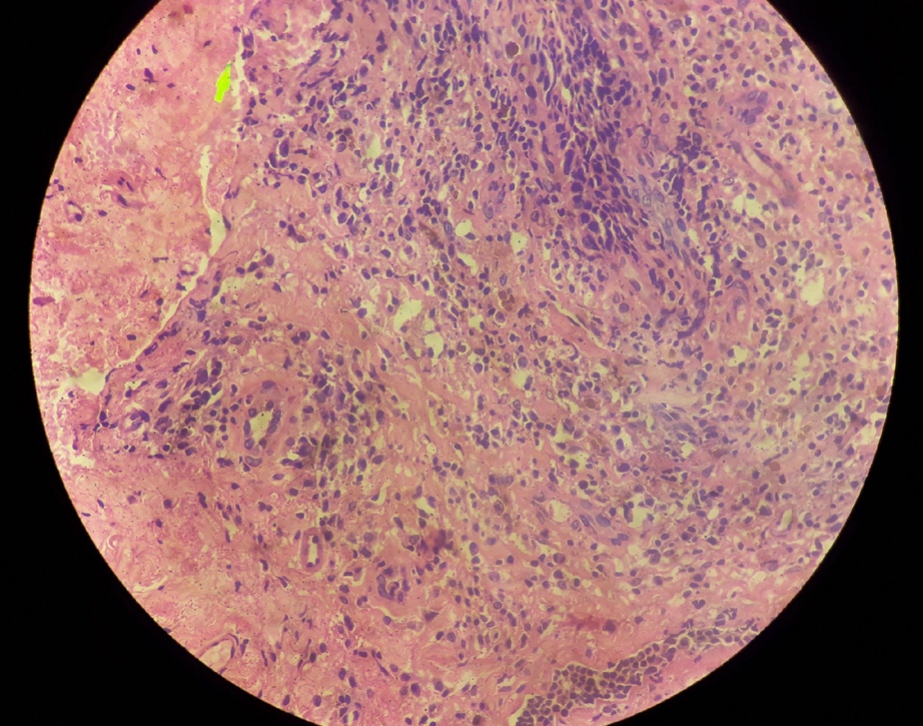
Hematoxylin and eosin stain, (100X). Histopathology showing chronic inflammatory infiltrate comprising plasma cells, lymphocytes, and macrophages in the dermis.

In the examination, mild tenderness was also noted. A punch biopsy from a lesion was taken. Histopathological examination revealed the presence of chronic inflammatory cells consisting of plasma cells, lymphocytes, and hemosiderin-laden macrophages along with scattered capillary-sized blood vessels without any atypia consistent with plasma cell vulvitis (Figure 2).

**Figure 3:**
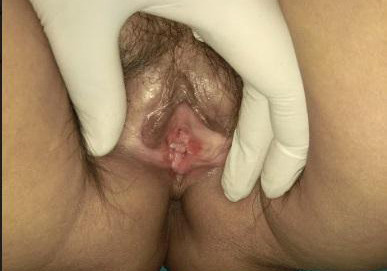
Marked reduction in erythema after 6 weeks of the first session of PRP.

The patient was treated with clobetasol propionate 0.05% cream, tacrolimus 0.1% ointment, and cryotherapy. However, the patient's symptoms did not improve. So, the patient was scheduled for PRP, 3 weeks after the last cryotherapy session. PRP was prepared by the double-spin open method. First, 17 ml of blood was withdrawn from the patient in 2 Acid Citrate Dextrose Solution A vacutainer tubes. The first centrifugation was done at 100 gm for 10 minutes, after which red blood cells were sedimented on the bottom. The supernatant plasma was centrifuged at 400 g for 15 minutes. The upper two-thirds of platelet-poor plasma was discarded, and approximately 2.5 mL of PRP was obtained. Then PRP was injected into the lesions and repeated after 2 weeks. The symptoms improved significantly within 2 weeks of the first session and resolved entirely at 6 weeks' follow-up. There were no side effects with PRP therapy during the procedure or on follow-up. The lesions also become less visible as shown in (Figure 3).

## DISCUSSION

In 1952, Zoon described a chronic inflammatory condition of the glans penis, or inner surface of the foreskin, clinically similar to erythroplasia of Queyrat called balanitis chronica circumscripta plasmacellularis, histologically characterised by plasma cell infiltrate. A similar condition in women known as Zoon vulvitis was described in 1954.^2^ The aetiology of plasma cell vulvitis is unknown, though autoimmune aetiology has been suspected along with hormonal, infectious, and irritant factors. Due to the rarity of this condition, information about the clinical course is limited.^3^ The most common symptom is a burning sensation, followed by dyspareunia, itching, soreness, and irritation. Our patient presented with itching, pain, and irritation. Other symptoms reported are dysuria, vaginal discharge, bleeding, dryness, and incontinence. Clinically, it presents as shiny, atrophic erythematous red to 'cayenne pepper' like plaques, and well-demarcated macules can be seen. It commonly involves the vulvar vestibule, periurethral area, and labia minora. A systematic review found that the most common presentation was erythema followed by erosions and less commonly with ulcerations.^3^ Erosive lichen planus and pemphigus are clinical differential diagnoses of plasma cell vulvitis and can be differentiated by histopathological examination with characteristic findings of a dense infiltrate largely composed of plasma cells in the dermis, which is similar and consistent with the findings in our case too.

A case report by immunohistochemistry showed a dense dermal infiltrate of plasma cells (CD138+) and also a mixed T/B infiltrate (CD3+/CD20+).^4^ The immunohistochemistry analysis of the specimen could not performed due to unavailability. Plasma cell vulvitis may be recalcitrant to the most commonly used topical corticosteroid and topical tacrolimus.^3^ Other treatment options with limited efficacy are imiquimod, oestrogen, misoprostol, interferon-alpha, cryotherapy, CO_2_ laser, PRP, and surgical resection.^1,3-5^ Due to repeated treatment failures with topical steroids, immunomodulators, and cryotherapy, we opted for PRP to treat the patient. Platelets are a good source of growth factors like platelet-derived growth factor, insulin-like growth factor, vascular endothelial growth factor, and transforming growth factor-beta.6 These growth factors inhibit NF-kB and suppress pro-inflammatory cytokines like COX-2, and chemokine receptors like CXCR4, thus reducing inflammation.^6-7^ In a similar case, reported improvement of plasma cell vulvitis after PRP therapy, a single case reported to date in the literature.^4^

Being a rare case of vulvar inflammation, one needs to explore all the possibilities for the diagnosis as well as management of unresponsive cases. There are no proper guidelines for the management of plasma cell vulvitis and physicians may need to explore different options available.

Plasma cell vulvitis, which is a rare condition with limited treatment modalities, is associated with treatment failures, hampering the quality of life, as seen in our patient. PRP may be a promisable therapeutic option. Further prospective large-scale studies with long-term follow-up should be carried out for this novel treatment option for Plasma cell vulvitis. The patient was satisfied with the treatment both symptomatically and clinically it improved with treatment with PRP therapy after multiple treatment modalities failed.
